# Applying a Fluorescence Polarization Assay for Detection of Brucellosis in Animals Using the Fluorescently Labeled Synthetic Oligosaccharides as Biosensing Tracer

**DOI:** 10.3390/bios14080404

**Published:** 2024-08-21

**Authors:** Liliya I. Mukhametova, Dmitry O. Zherdev, Sergei A. Eremin, Anton N. Kuznetsov, Viktor I. Yudin, Oleg D. Sclyarov, Olesia V. Babicheva, Anton V. Motorygin, Yury E. Tsvetkov, Vadim B. Krylov, Nikolay E. Nifantiev

**Affiliations:** 1 Department of Chemistry, Moscow State University, 119991 Moscow, Russia; liliya106@mail.ru (L.I.M.); dmitryzher@yandex.ru (D.O.Z.); 2 N.D. Zelinsky Institute of Organic Chemistry, Russian Academy of Sciences, 119991 Moscow, Russiavictor12yudin@yahoo.com (V.I.Y.); tsvetkov@ioc.ac.ru (Y.E.T.); 3 Russian State Centre of Quality and Standardization of Veterinary Drugs and Feeds, 123022 Moscow, Russia; odsklyarov@vgnki.ru (O.D.S.); a.babycheva@vgnki.ru (O.V.B.); avmotorygin@vgnki.ru (A.V.M.)

**Keywords:** *Brucella*, O-antigen, N-formyl-d-perosamine, antibodies detection, fluorescence polarization assay

## Abstract

Brucellosis in animals is an infectious disease caused by bacteria of the genus *Brucella*. Known methods for diagnosing brucellosis face some challenges, due to the difficulties in isolating and standardizing the natural brucellosis antigen. In this work, we investigated the possibility of using the fluorescence polarization assay (FPA) with synthetic glycoconjugate biosensing tracers to detect antibodies against *Brucella* as a new methodology for diagnosing brucellosis. Based on the received results, the synthetic fluorescein-labeled trisaccharide tracer is most effective for Brucellosis detection. This tracer is structurally related to the immune determinant fragment of the *Brucella* LPS buildup of N-formyl-d-perosamine units, connected via α-(1→3)-linkage at the non-reducing end and α-(1→2)-linkage at the reducing end. The sensitivity and specificity in the case of the use of trisaccharide tracer **3b** were 71% and 100% (Yuden’s method) and 87% and 88% (Euclidean method), respectively, which is comparable with the diagnostic efficiency of traditionally used serological methods, such as the agglutination test (AT), complement fixation test (CFT), and Rose Bengal test (RBT). Given the known advantages of FPA (e.g., speed, compactness of the equipment, and standard reagents) and the increased specificity of the developed test system, it would be appropriate to consider its widespread use for the diagnosis of brucellosis in animals, including rapid testing in the field.

## 1. Introduction

Brucellosis is an infectious-allergic zoonotic disease caused by bacteria of the genus *Brucella* [1]. Infection of humans and animals most often occurs through direct contact with sick animals, raw materials of animal origin (e.g., wool, fluff, and skins), animal care items, and other objects infected by *Brucella*. The alimentary route of infection is also possible through the consumption of animal products contaminated with *Brucella* (e.g., milk and meat products) [2]. Currently, nine species of *Brucella* belong to the second group of pathogenicity. Among these, three types of pathogens are of the greatest epizootiological and epidemiological importance: *B. melitensis*, the reservoirs of which are goats and sheep; *B. abortus*, its carriers are cattle; and *B. suis*, the main carriers of which are pigs, as well as, additionally, hares, reindeer, and mice [1]. Early diagnosis defines the success of prevention and control of the spread and elimination of brucellosis. Currently, several methods are used to detect this disease in animals. In particular, direct detection of *B. abortus* is achieved using bacteriological examination or polymerase chain reaction (PCR) [3]. Although bacterial cell culture and biochemistry are considered the gold standard for confirming *B. abortus* infection, the test results can be affected by various factors, such as bacterial viability, tissue concentration, environmental contamination, and others [4,5]. These methods also have a number of disadvantages, including high cost and long analysis times, and, therefore, are not suitable for out-of-laboratory disease screening [6].

Serological tests, which detect specific antibodies to the pathogen, are superior to methods based on cell culturing and isolation of bacteria because of simplicity, speed of execution, and low cost. In addition, these methods are characterized by relatively high diagnostic sensitivity and specificity [7,8,9], which leads to their widespread use for the detection of brucellosis in animals and humans. Serological tests are particularly valuable for diagnosing animals with clinical indications of the disease. The detectable levels of IgM antibodies against *B. abortus* in dairy cattle are usually achieved one week after infection, whereas IgG antibodies become detectable in two weeks after infection and persist throughout life [10]. Described diagnostic protocols have an essential disadvantage due to the use of the O-antigenic part of the appropriate *Brucella* lipopolysaccharide (LPS). The biotechnological cultivation and subsequent release, as well as the purification of the O-antigenic chain followed by its functionalization, are very laborious and may result in unreproducible polysaccharide samples. The RBT and SAT are commonly used in epidemiological studies to estimate the prevalence of *B. abortus* in dairy cattle [11]. Although RBT and SAT are considered accurate, their results are assessed visually and may be subjective and dependent on the skills and experience of the operator [7]. This justifies the need to develop more reliable tests for the diagnostics of brucellosis, especially those that allow an automated assessment of the test result.

In this paper, we report on the use of the fluorescence polarization assay (FPA) in combination with fluorescently labeled synthetic oligosaccharide biosensing tracers for the detection of antibodies against *Brucella* as a new option for brucellosis serodiagnosis. FPA is a rapid assay platform technology that allows rapid and accurate detection of specific antibodies or antigens [12]. The FPA principle has been widely used to detect biological interactions, particularly involving antigen–antibody binding [13], as well as enzymes and their substrates [14,15]. FPA meets the standards of the World Organization for Animal Health, and is recommended as a method for laboratory testing of animal brucellosis [16,17]. In addition to the short testing time, another advantage of FPA is that it produces quantitative results through automated reading and electronic archiving. They eliminate the subjectivity of the results and allow their dissemination for serial diagnostic studies, including large-scale field screening for the monitoring of possible infectious lesions. A number of studies have focused on the development of the FPA technology for the detecting of antibodies against various *Brucella* species in physiological fluids (e.g., serum, whole blood, milk) of cattle [18], sheep [19], pigs [20], deer [21], camels [22], and humans [23,24]. In these studies, fluorescein (FITC)-labeled O-antigen was used as a tracer, which was obtained after chemical cleavage of the *B. abortus* S1119.3 lipopolysaccharide and separation of the O-polysaccharide from the lipid A [25]. Although it has been shown that the use of tracers based on the O-polysaccharide from *B. abortus* S1119.3 enables the detection of anti-*Brucella* antibodies in cattle blood or milk [26], these tracers are not practical due to the disadvantages described above. They are associated with the non-standard nature of the native O-polysaccharide chains and uncertainties of their transformation into FITC-labeled conjugates.

**Figure 1:**
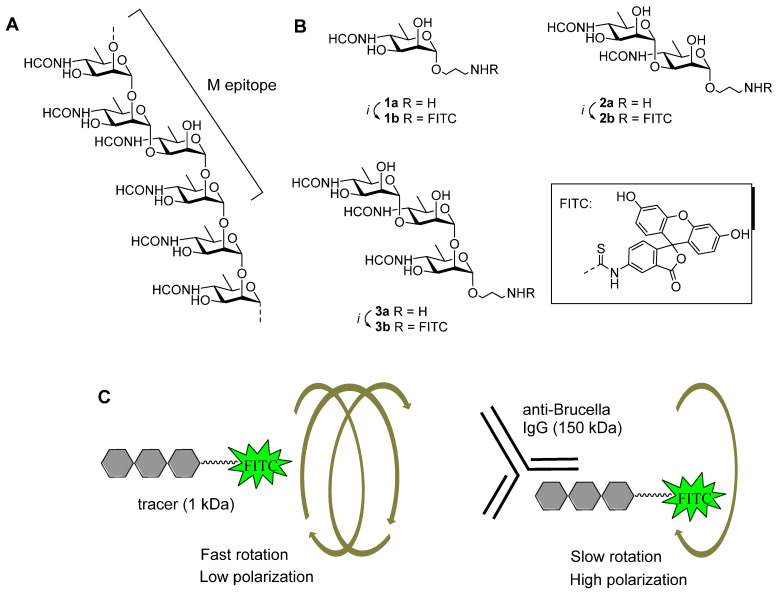
(**A**). Structure of the *Brucella* O-polysaccharide showing the M epitope. (**B**). Synthesis of FITC-labeled biotracers **1b**–**3b** used in FPA diagnostic tests. Reagents and conditions: *i*: FITC (1.2 eq), Na_2_CO_3_ (3 eq), DMF, H_2_O, 60 °C. (**C**). The principle of FPA detection of brucellosis.

To overcome these limitations, we explored the possibility of replacing tracers based on O-polysaccharides with FITC-labeled short synthetic oligosaccharide derivatives **1b–3b** (Figure 1). Such glycoconjugate biosensing derivatives of different structures relate to defined immunologically relevant fragments of the O-polysaccharide chain of *Brucella* LPS. Compounds **1b–3b** also contain a FITC tag connected via a short aglycone spacer. This improvement in the protocol became possible due to modern methods of carbohydrate chemistry, which now permit the efficient and reproducible preparation of practically any oligosaccharide derivative [27] required for raising related antibodies [28] and characterization of their specificity [29], for the development of sensitive and specific diagnostic test systems to detect carbohydrate antigens and corresponding antibodies.

## 2. Materials and Methods

### 2.1. Reagents and Equipment

Synthetic spacer-armed mono-, di-, and trisaccharide derivatives **1a** [30], **2a** [31], and **3a** [30] (Figure 1) related to the O-antigens of *Brucella* spp. were obtained as previously described. Commercially available fluorescein isothiocyanate was used for labeling. Isolation of conjugates was carried out on a reversed-phase Sep-Pak C18 cartridge. High-resolution mass spectra were obtained using a Bruker micrOTOF II spectrometer with electrospray ionization (ESI). Both positive ion (capillary voltage of −4500 V) and negative ion spectra (capillary voltage of 3200 V) were recorded. The used mass range (*m*/*z*) was 50–3000 Da; external and internal calibrations were applied (Electrospray Calibrant Solution, Fluka). Solutions of the analytes in acetonitrile, methanol, or water were injected through a syringe injector at a flow rate of 3 μL min^−1^. Nitrogen was used as a nebulizer gas (4 L min^−1^); interface temperature was 180 °C. Fluorescence intensity and mP were measured using a portable fluorescence polarization reader Sentry-200 (Ellie LLC, Germantown, WI, USA) in a borosilicate glass tube (10 × 75 mm). LED and a photomultiplier tube were used as the light source and detector, respectively; λex = 485 nm and λem = 535 nm.

### 2.2. Synthesis of Fluorescein-Labeled Oligosaccharide Tracers 1b–3b

FITC was conjugated to synthetic oligosaccharides **1a**–**3a** as described previously [14]. Briefly, aminopropyl glycosides **1a**–**3a** (1 eq.) were treated with fluorescein isothiocyanate (1.5 eq.) in a water/DMF mixture in the presence of Na_2_CO_3_. The obtained FITC-labeled conjugates **1b**–**3b** were isolated using a reversed-phase Sep-Pak C18 cartridge. The preliminarily washed cartridge was eluted with 2 mL portions of methanol–water mixture (from 0 to 60 vol% of methanol) with the concentration increasing in increments of 5 vol%. Product **1b** was collected at eluent concentrations between 30 and 45 vol%, products **2b** and **3b** between 15 and 30 vol%. After evaporation and lyophilization, the products **1b**–**3b** were obtained as light orange fluffy solids in yields of 62%, 80%, and 77% respectively. The purity of the products was confirmed by thin-layer chromatography. High-resolution mass spectrometry (HRMS ESI): calcd. for **1b** C_31_H_31_N_3_O_10_S [M+Na]^+^ 660.1622 found 660.1613; calcd. for **2b** C_38_H_42_N_4_O_14_S [M+Na]^+^ 833.2310 found 833.2304; calcd. for **3b** C_45_H_53_N_5_O_18_S [M+Na]^+^ 1006.2999 found 1006.2989.

### 2.3. Serum Samples

Bovine serum (110 samples) was obtained from the collection of the All-Russian State Center for Quality and Standardization of Drugs for Animals and Feeds. Beforehand, all serum samples were tested using RBT, CFT, and ELISA in parallel, and divided into two groups.

(a) Brucellosis positive (N = 93)—serum samples from several brucellosis-unfavorable farms with a positive reaction in at least two of the used serological tests.
(b) Brucellosis negative (N = 17)—the reaction to all serological tests was negative for brucellosis.

### 2.4. Fluorescence Polarization Assay

Tracer working solutions of FITC-labeled glycoconjugates **1b**–**3b** (2.5 nM) in 10 mM phosphate buffer containing 0.15 M NaCl, pH 7.4, were prepared in such a way that the fluorescence intensity of the solutions was 10 times higher than the background signal of buffer and amounted to about 200,000 U. Then, tested serum (10 µL) was added to the tracer working solution (1.0 mL), the tube was vigorously stirred, incubated for 1–2 min at 20 °C and fluorescence polarization was measured. All measurements were performed in triplicate.

### 2.5. Data Analysis

For each serum sample, the fluorescence polarization measurements were repeated three times and presented as an average value. The coefficient of variation between repeated measurements did not exceed 0.1. The cutoff value, the selectivity, the specificity, and the area under the curve (AUC) were determined with ROC analysis using the statistical program Sigma Plot 11 software package (Systat Software Inc., San Jose, CA, USA).

## 3. Results and Discussion

Among the tested compounds the oligosaccharide derivatives **2b** and **3b** (Figure 1B) contain an α-(1→3)-glycosidic bond between perosamine residues as in the M-epitope of the *Brucella* O-polysaccharide [32,33] (Figure 1A). This structural element is specific for O-antigens of *Brucella*, but not for other bacteria producing perosamine-containing polysaccharides (e.g., *Yersinia enterocolitica*, *Escherichia coli*, *Vibrio cholerae*) and which may interfere with the serological diagnosis of brucellosis.

**Figure 2:**
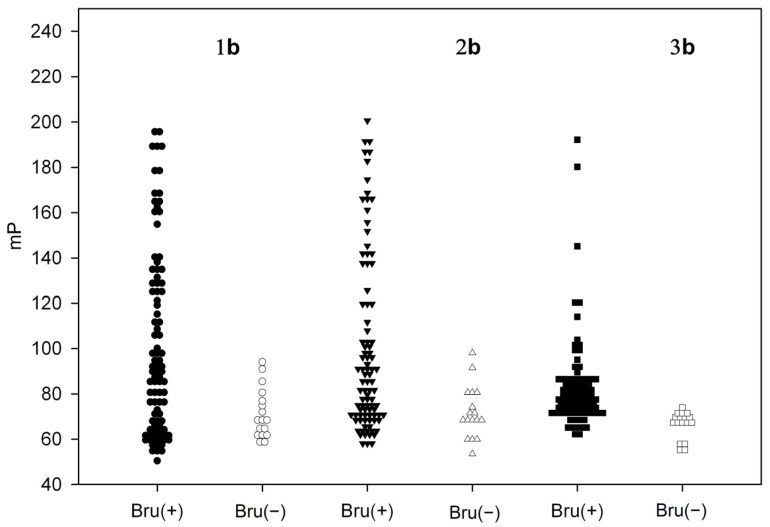
The results of FPA of Bru(+) (N = 93) and Bru(−) (N = 17) brucellosis bovine serum samples using tracers **1b–3b**.

Fluorescently labeled tracers in the unbound state emitted depolarized light due to their rapid rotation. When the resulting oligosaccharide tracers bind to anti-brucellosis IgG, circulating in the sera of infected animals, the degree of polarization (mP) increases due to restricted rotational diffusion of the high-molecular-weight immune complex. FPA of bovine serum samples was carried out using the obtained tracers **1b**–**3b** (Figure 2). Based on the analysis of the test results obtained with reference methods (RBT, CFT, ELISA), 93 serum samples from various farms infected with brucellosis were accepted as positive and denoted as Bru(+); 17 serum samples from non-endemic regions, for which all reference tests performed were negative, formed a negative group and denoted as Bru(−). For all three tracers (**1b**–**3b**), the spread and median fluorescence polarization values were significantly higher for Bru(+) samples (Figure 2, Table 1), confirming their ability to interact with specific anti-*Brucella* bovine immunoglobulins. It is interesting to note that as the oligosaccharide length increased, the median fluorescence polarization of positive samples decreased (Table 1). It can be due to the increased distance between the FITC label and the fragment recognized by antibodies.

**Figure 3:**
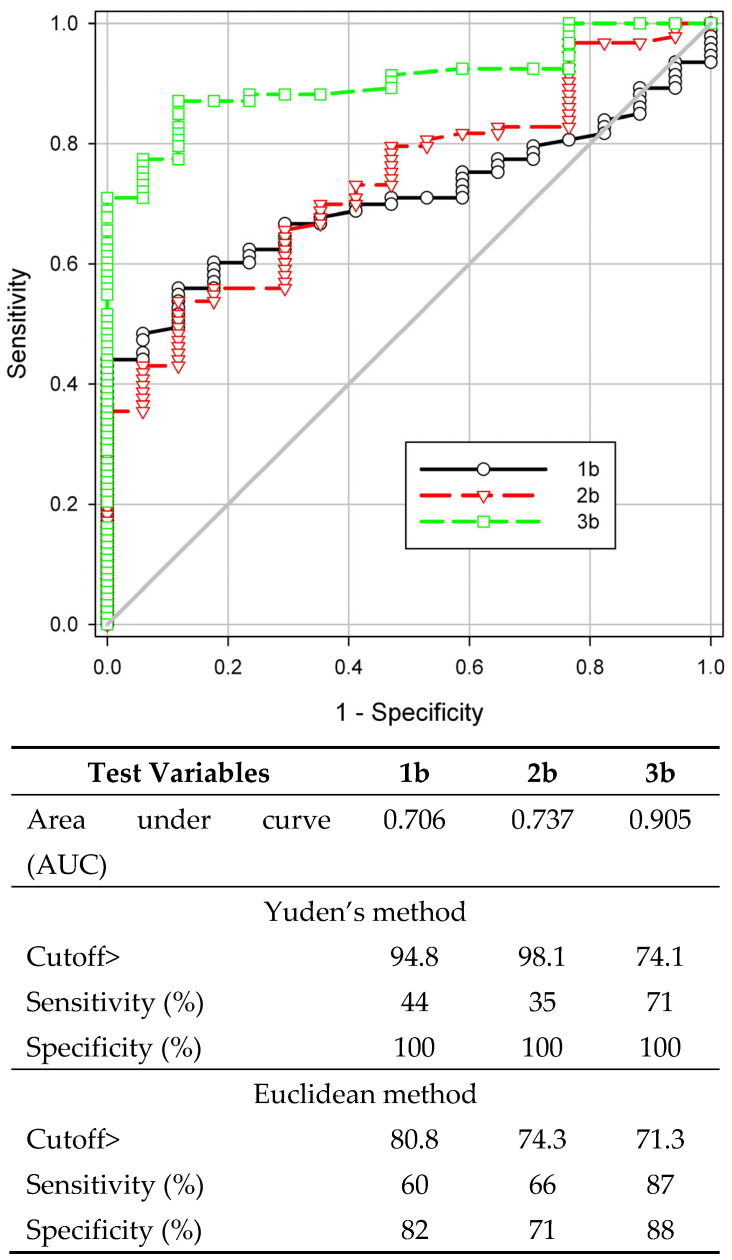
Receiver operating characteristic (ROC) analysis of sensitivity (%) plotted against 1—specificity (%) to determine the optimal cutoff value of fluorescence polarization immunoassay (FPA) for the detection of antibodies against *Brucella* by FPA for tracers **1b**–**3b**. The characteristics of the ROC curves calculated by Yuden’s and Euclidean methods are presented in the table below.

To determine the diagnostic characteristics and select the optimal biosensing tracer, an ROC analysis of the obtained data was performed (Figure 3). The area under the ROC curve (AUC) increased with the lengthening of the oligosaccharide chain and was 0.706, 0.737, and 0.905 for mono-, di-, and trisaccharide derivatives **1b**–**3b**, respectively. The diagnostic sensitivity determined by the Youden method was 45, 35, and 71%, respectively, with a specificity of 100%. Thus, among the compounds studied, the trisaccharide tracer **3b** had the best diagnostic performance and is suitable for detecting brucellosis in cattle. The cutoff value calculated using the Euclidean method was slightly lower than that obtained using Yuden’s method; the diagnostic sensitivity and specificity were 87% and 88%, respectively. It is important to note that, in this case, the results of the determination were comparable with the efficiency of classical serological methods—AT, CFT, and RBT (Table 2).

## 4. Conclusions

In conclusion, we developed the synthetic FITC-labeled biosensing trisaccharide tracer **3b** with a strictly defined structure for the detection of brucellosis in cattle using the fluorescence polarization method. The trisaccharide tracer **3b** demonstrated comparable diagnostic efficiency to traditionally used serological methods, such as AT, CFT, and RBT, although the shorter tracers **1b** and **2b** were not effective. The described protocol based on FPA has valuable competitive advantages including its short performance time, compact equipment, standard reagents, and the ability to instrumentally record results. The oligosaccharide tracer **3b** described here offers several advantages over previously described [18,19,20,21,22,23,24] tracers based on natural O-polysaccharide antigens. These advantages include (a) the absence of the need to work with *Brucella* bacterial culture to make an O-polysaccharide-based tracer; (b) the use of a synthetic tracer with a well-defined and reproducible structure, which is also free of interfering microbial impurities; and (c) the presence of an aglycon linker in synthetic oligosaccharide chain permits the site-specific introduction of a fluorescent tag. These advantages greatly simplify tracer standardization and lead to obtaining reproducible analysis results. This demonstrates a good potential for the practical application of the developed diagnostic method in animal diagnosis.

## Figures and Tables

**Table 1:** Medians, means, and standard deviation of fluorescence polarization (mP) of Bru(+) and Bru(−) bovine serum samples registered with the use of tracers **1b**–**3b**.

Characteristic	Group	Fluorescence Polarization (mP) for Tracers		
		1b	2b	3b
Range	Bru(+)	50.5–195.7	57.2–200.6	62.2–192.2
	Bru(−)	58.2–94.1	53.5–98.0	54.5–74.0
Median (LQ–UQ *)	Bru(+)	89.4 (65.4–128.8)	85.6 (70.7–119.8)	78.0 (72.8–85.4)
	Bru(−)	68.2 (62.3–76.9)	69.0 (68.0–80.0)	68.0 (66.5–70.6)

* LQ, lower quartile; UQ, upper quartile.

**Table 2:** Results of a comparative study of blood serum samples from cattle in AT, CFT, RBT, and FPA. The number of sera samples identified as positive, questionable, and negative is presented.

Total	Results	AT	CFT	RBT	FPA 1b	FPA 2b	FPA 3b
110	Positive	75	87	84	59	66	83
	Questionable	11	0	-	-	-	-
	Negative	24	23	26	51	44	27

## Data Availability

All data generated or analyzed during this study are included in this published article. Any additional data will be available upon request.
